# Male Breast Cancer in a Bronx Urban Population: A Single-Institution Retrospective Observational Study

**DOI:** 10.3390/diagnostics16020339

**Published:** 2026-01-21

**Authors:** Kristen Lee, Bhakti Patel, Ruth Samson, Emily Hunt, Christian L. Sellers, Takouhie Maldjian

**Affiliations:** 1 Department of Radiology, Montefiore Medical Center, Albert Einstein College of Medicine, Bronx, NY 10461, USAemily.hunt@einsteinmed.edu (E.H.); 2 Department of Breast Surgery, Montefiore Medical Center, Albert Einstein College of Medicine, Bronx, NY 10461, USA; rsamson@montefiore.org; 3 School of Medicine, Meharry Medical College, Nashville, TN 37208, USA; christian.sellers@mmc.edu

**Keywords:** gynecomastia, mammogram, male breast cancer, underserved population

## Abstract

**Background/Objectives**: This study seeks to evaluate the clinical characteristics of newly diagnosed male breast cancers within the traditionally underserved Bronx population at risk for poorer health outcomes. **Methods**: We retrospectively searched our database for male patients who presented for mammographic evaluation between 1 January 2016 and 1 October 2024. The primary outcomes were the prevalence of biopsy-proven male breast cancer and its association with gynecomastia and TNM stage at diagnosis. Clinical data, including TNM staging, receptor status, risk factors, and patient demographics, were recorded for patients with biopsy-proven breast cancer based on biopsy results. Two dedicated breast imagers retrospectively evaluated mammograms of these patients to determine by consensus the presence of gynecomastia. Analyses were descriptive in nature. **Results**: During the study period, 423 screening mammograms and 1775 diagnostic mammograms were performed on male patients. Twenty-six male patients with biopsy-proven breast cancer were identified (two were bilateral and four were multifocal). In total, 69% of our male breast cancer patients (18 out of 26) demonstrated gynecomastia, which was similar across demographic groups, ranging from 63 to 75%. Out of the three patients with Stage 4 disease, two were Black and one was White. Stage 3 or higher disease was seen in 29% of our Black patients, 12% of our White patients, and 0% of our Hispanic patients. **Conclusions**: Male breast cancer in this Bronx population was frequently associated with gynecomastia and showed notable demographic disparities. Black patients presented with more advanced disease than other demographic groups. These descriptive findings highlight areas of further investigation and may help inform future outreach and early detection efforts in high-risk, underserved communities. This retrospective, single-institution analysis was limited by a small sample size and did not include formal statistical testing; therefore, the findings are descriptive and warrant validation with larger cohorts.

## 1. Introduction

Male breast cancer is a rare disease, and due to its low incidence, it remains understudied, and clinical management is largely extrapolated from data derived from female breast cancer populations. Established risk factors include genetic predisposition (particularly BRCA2 mutations), hormonal imbalance, obesity, liver disease, radiation exposure, and conditions associated with hyperestrogenism. Gynecomastia, a common benign condition in men, frequently coexists with male breast cancer, although its clinical significance as a risk marker remains incompletely defined. Prior studies suggest a potential association between gynecomastia and increased male breast cancer risk, though evidence is limited. In this study, we aim to characterize the presence of gynecomastia among males diagnosed with breast cancer at our institution and explore whether it is commonly observed across demographic groups. While this is a descriptive, hypothesis-generating investigation, focusing on gynecomastia allows us to evaluate a potentially important clinical and imaging feature in an underserved population at risk for advanced disease. Men with breast cancer often present at a more advanced stage than women, largely due to the absence of routine screening, limited awareness, and delays in diagnosis. Racial and ethnic disparities have been reported. Invasive breast cancer is more prevalent and demonstrates a poorer prognosis in Black men relative to men from other racial groups [1,2]. However, data examining male breast cancer in racially diverse and underserved urban populations remain limited. Large registry-based and epidemiologic studies have characterized incidence and survival trends; however, these datasets lack imaging-level detail and do not allow for radiologist-adjudicated assessment of mammographic findings such as gynecomastia. Unlike registry-based or epidemiologic studies, this analysis incorporates dedicated radiologist review of mammograms to assess gynecomastia and directly correlate imaging findings with TNM stage and patient demographics in an underserved population. Additionally, underserved urban populations, including those in the Bronx, experience disproportionate health disparities that may influence stage at presentation and outcomes in male breast cancer. The Bronx houses a population especially prone to poor health outcomes, given the high prevalence of chronic disease and health care disparities, and the South Bronx especially includes many Black communities predisposed to higher morbidity and mortality for many diseases, including male breast cancer. The primary objective of this study was to describe the prevalence and clinicodemographic characteristics of newly diagnosed male breast cancer in an underserved Bronx population. Secondary objectives were (1) to evaluate the prevalence of mammographically identified gynecomastia among men with biopsy-proven breast cancer, and (2) to examine the distribution of TNM stage at diagnosis across demographic groups. By incorporating dedicated radiologist review of mammograms, this study provides imaging-level insights not available in registry-based analyses. This descriptive, single-institution case series aims to generate hypothesis-generating data that may inform future studies focused on early detection and disparities in male breast cancer within underserved communities. By examining associations with gynecomastia, tumor stage at diagnosis, and demographic factors, this study aims to provide descriptive insights that may inform future outreach, early detection efforts, and public health strategies in high-risk communities.

## 2. Materials and Methods

Institutional Review Board (IRB) and Health Insurance Portability and Accountability (HIPAA) approval were obtained. Informed consent was waived by the IRB. This study was a single-institution, retrospective descriptive case series of male patients who underwent mammographic evaluation between 1 January 2016 and 1 October 2024 and were diagnosed with biopsy-proven breast cancer, identifying 26 patients. The study period was selected to capture an adequate number of cases of this rare malignancy while ensuring uniform imaging protocols and diagnostic practices throughout the study interval. After reviewing each case, information was recorded regarding the presence or absence of gynecomastia, hormone receptor characteristics of each tumor, tumor size and stage, multifocality or multicentricity, bilaterality, risk factors, and ethnic/racial demographics. The presence of gynecomastia was determined retrospectively by consensus of two board-certified breast radiologists who were aware of the diagnosis of breast cancer and the clinical context during assessment of gynecomastia, based on evaluation of each patient’s mammogram. Standard full-field digital mammography was performed using routine craniocaudal and mediolateral oblique views in accordance with institutional and MQSA guidelines [3]. On mammography, gynecomastia was defined as subareolar nodular opacity if nodular, subareolar linear opacity if dendritic, or parenchyma resembling a dense female breast if diffuse [4,5]. Gynecomastia was recorded as a binary variable (present/absent) based on consensus review by two dedicated breast imagers. Clinical data, including TNM stage, multifocal/multicentric disease, bilateral disease, receptor types, patient demographics, and overall survival, were recorded. Survival status was defined as alive or deceased at the last available clinical follow-up, as documented in the electronic medical record. The study population consisted of male patients undergoing mammographic evaluation at our institution during the study period. Analyses were limited to patients with biopsy-proven breast cancer within a referred clinical population and were not intended to estimate population-level disease prevalence and may not be generalizable to the community at large.

The limited sample size of 26 patients restricts statistical power and precludes formal hypothesis testing, increasing the risk of type II error and limiting generalizability. Given the small sample size and limited number of events within subgroups, the study was not powered to perform inferential statistical analyses, and formal hypothesis testing (including *p*-values, confidence intervals, or comparative statistics) was not conducted. The descriptive nature of the analyses is to highlight any observed differences between demographic groups for exploratory and hypothesis-generating purposes.

As a tertiary referral center within this community, our institution receives both internally referred patients and external referrals from affiliated clinics, which may contribute to later-stage presentation. Selection bias may be present, as our cohort includes only male patients who underwent mammographic evaluation at a single tertiary care institution, which may not be representative of all men with breast disease in the Bronx community, particularly those who did not seek care or were evaluated elsewhere. Referral bias is also possible, as patients referred for diagnostic mammography may have had more severe symptoms or higher clinical suspicion, potentially inflating the observed prevalence of malignancy and advanced-stage disease. Survivorship bias may have influenced staging distributions, as patients with rapidly progressive disease who did not survive to imaging evaluation or biopsy would not be captured in our dataset. Information bias is inherent in retrospective chart review, as clinical risk factors, staging data, and demographic variables were dependent on the completeness and accuracy of medical records. Misclassification bias is also possible, particularly in the assessment of gynecomastia, although this was mitigated by independent retrospective review of mammograms by two dedicated breast imagers with consensus agreement.

## 3. Results

**Figure 1:**
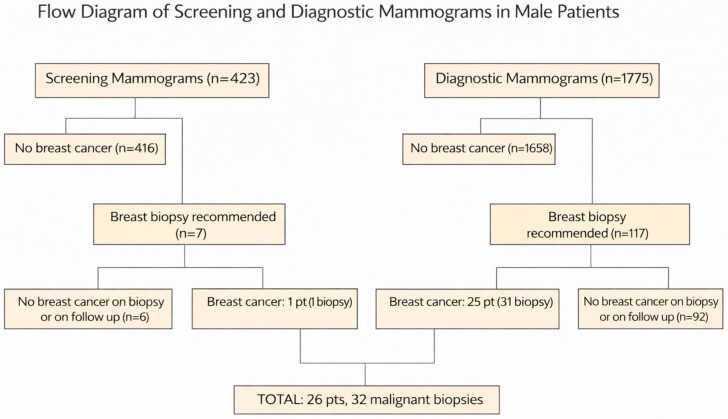
Flow diagram illustrating outcomes of screening and diagnostic mammograms in male patients. Two parallel pathways converge on a final cohort of 26 patients with biopsy-proven breast cancer, representing 32 malignant biopsies.

During the eight-year study period, a total of 2198 mammograms were performed, including 423 screening mammograms and 1775 diagnostic mammograms. Biopsy was recommended in 124 patients. Ninety-eight patients had no breast cancer on biopsy or follow-up. Thirty-two biopsies (in 26 patients) revealed breast cancer during this period, as seen in the flow diagram (Figure 1). Twenty-six male patients with breast cancer were identified over the eight-year period, with ages ranging from 43 to 90 years (Table 1), with a mean age of 67.7. Fourteen patients had left breast cancer, ten had right breast cancer, and two had bilateral breast cancer.

### 3.1. Receptor Types

All cancers were ductal, and all were estrogen receptor positive (ER+). Three patients also tested positive for HER2 and presented with T2N1, T4bN3M1, and T2N1a disease, all of which presented with locally advanced (N1) or metastatic (M) disease. Two of the HER2+ patients were Black, and one was Hispanic.

### 3.2. Demographics and Gynecomastia

**Figure 2:**
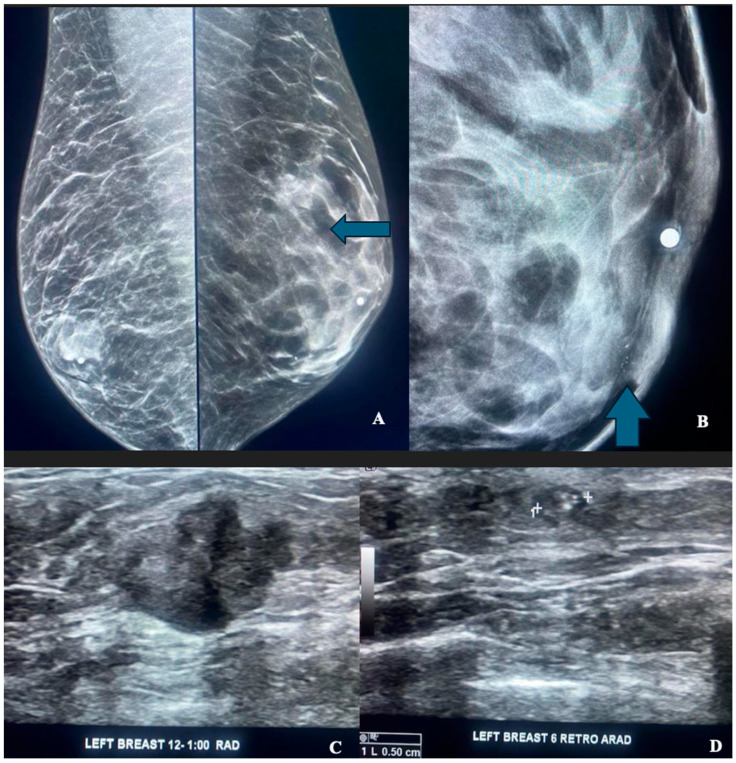
(**A**–**D**) Multifocal breast cancer of the left breast in a patient presenting with a palpable lump in the left breast. (**A**) Bilateral MLO view demonstrates bilateral gynecomastia, left greater than right, and a partially obscured mass (arrow) in the upper left breast within a background of dense breast tissue. (**B**) Calcifications at the left 6:00 axis anteriorly (arrow) are seen to better advantage on this magnification view. (**C**) Ultrasound delineates a suspicious hypoechoic mass at the left 12:00 to 1:00 axis, corresponding to a partially obscured mass in this region on mammography. Ultrasound-guided biopsy revealed invasive ductal carcinoma. (**D**) Ultrasound demonstrates an ovoid hypoechoic 5 mm nodule containing calcifications, corresponding to suspicious calcifications at the anterior left 6:00 axis on mammography. Ultrasound-guided biopsy revealed DCIS.

**Figure 3:**
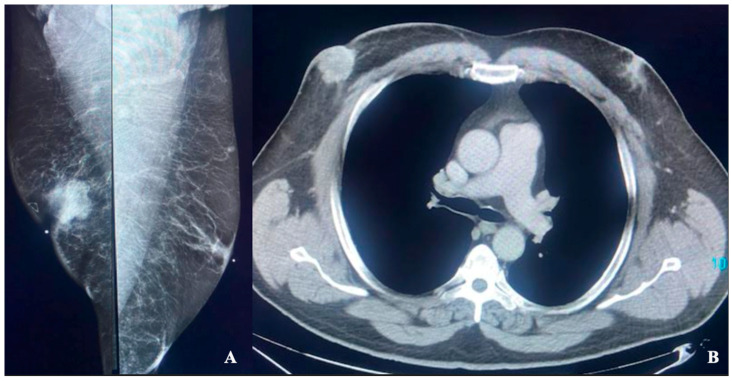
(**A**,**B**) Mass in the right breast and gynecomastia in a patient presenting with a palpable lump in the right breast. (**A**) Bilateral MLO view demonstrates a right retroareolar mass with nipple retraction and skin involvement at the site of the triangular skin marker denoting the location of the palpable lump. Biopsy revealed invasive ductal carcinoma. Subareolar linear opacities are seen on the left consistent with dendritic gynecomastia. (**B**) Axial CT of the chest demonstrates a right retroareolar mass, which was demonstrated to be breast cancer at biopsy. On the left, retroareolar breast tissue measures over 2 cm, consistent with gynecomastia.

Fourteen patients were Black, four were Hispanic, and eight were White. Eighteen of these twenty-six patients (69%) showed gynecomastia (Figure 2) based on the consensus of two board-certified breast radiologists. One patient also had a CT scan, which confirmed the presence of gynecomastia by CT criteria, defined by a maximal diameter of parenchymal tissue exceeding 2 cm (Figure 3) [6]. By demographic group, 10 of 14 (71%) Black males, 3 of 4 (75%) Hispanic males, and 5 of 8 (63%) White males had gynecomastia.

### 3.3. Demographics and Survival

Mean age at diagnosis differed across racial groups, measuring 70.8 for Blacks, 55.5 for Hispanics, and 68.3 for Whites. At the last known follow-up, 20 of the 26 patients were alive. Survival status by race at last follow-up included 10 out of 14 (71%) Black patients, 4 out of 4 (100%) Hispanic patients, and 6 out of 8 (75%) White patients (Table 1). Follow-up duration varied across patients, and these data are presented descriptively without formal time-to-event analysis. Three patients had metastatic disease at diagnosis, consisting of 2 out of 14 Black patients and 1 out of 8 White patients (Table 1). Of patients diagnosed over 5 years ago, a greater proportion of Hispanic males demonstrated 5-year survival compared to the other groups, with survival in all three patients diagnosed over 5 years ago. Four out of five Black patients and one out of three White patients diagnosed over 5 years ago survived. Due to the small sample sizes and variable follow-up, no meaningful statistical or comparative analysis can be performed. Therefore, these observations are intended to be interpreted descriptively and not as a comparative analysis.

### 3.4. Demographics and Staging

A total of 4 patients had a T3 or higher T-stage, including 3 out of 14 (21.4%) Black patients and 1 out of 8 (12.5%) White patients. Twelve patients had nodal involvement, comprising 8 out of 14 (57%) Black patients, 3 out of 8 (37.5%) White patients, and 1 out of 4 (25%) Hispanic patients (Table 1). In our cohort of 14 Black patients, there were 2 (14%) patients with Stage 4 disease, 2 (14%) with Stage 3C disease, 3 (21%) with Stage 2B disease, 2 (14%) with Stage 2A disease, 4 (28%) with Stage 1A disease, and 1 (7%) with Stage 0 disease. Among the 8 White patients, there was 1 (12%) patient with Stage 4 disease, 1 (12%) patient with Stage 2B, 2 (25%) patients with Stage 2A disease, 3 (37%) patients with Stage 1A, and 1(12%) patient with Stage 0. Of the 4 Hispanic patients, 2 (50%) had Stage 2B disease, 1 (25%) had Stage 2A, and 1 (25%) had Stage 1A. Of the three patients with Stage 4 disease, two were Black and one was White. Stage 3 or higher disease was observed in 29% of our Black patients, 12% of our White patients, and none of our Hispanic patients.

### 3.5. Multifocal, Multicentric, and Synchronous Disease

Four patients had multifocal disease, and none had multicentric disease. Two patients had synchronous bilateral disease, with one having unilateral multifocal disease. Multifocal and bilateral disease was observed more frequently in Black patients, accounting for 80% of those patients with multifocal and/or bilateral disease (Table 2A,B). Both patients with bilateral disease (100%) and three out of four patients with multifocal disease (75%) demonstrated gynecomastia (Table 1).

### 3.6. Risk Factors

Four patients had genetic mutations associated with a high risk: three with BRCA2+, one with CHEK2+, one with TP53 and ATM mutations, and one with a germline pathogenic variant of RAD51C. Among patients without a predisposing genetic mutation, at least 4 had a first-degree relative (daughter, mother, sibling) with breast cancer. In total, 20 of the 26 patients had genetic testing. A pathogenic mutation was found in 6 of the 20 patients tested, or 30%. If pathogenic mutation variants of uncertain significance are included, then there are 9 of the 20 patients with genetic mutation, or 45%. In total, 10 out of 14 Black patients were tested (71%), with two pathogenic mutations out of 10 patients tested (20%) and two mutations of uncertain significance, or four mutations out of 10 patients tested (40%). All four Hispanic patients (100%) were tested, revealing two pathogenic mutations of the four patients tested (50%) and one mutation variant of uncertain significance, or three mutations of the four patients tested (75%). Six out of eight White patients (75%) were tested, revealing two pathogenic genetic mutations in the six patients tested (33%). Additional risk factors and comorbidities are listed in Table 1.

## 4. Discussion

Male breast cancer is a rare but clinically significant malignancy with well-documented racial disparities in incidence, stage at presentation, and survival outcomes. Based on data from 11,990 men with invasive breast cancer from the U.S. Cancer Registry from 2010 to 2016, Black men demonstrated a 52% higher incidence compared to non-Hispanic white men [1]. Additionally, mounting evidence shows that African American men with breast cancer have worse survival outcomes [2]. This trend toward diminished survival in Black male patients with breast cancer reflects the same disparity in survival rates among Black women with breast cancer compared to other groups of women [7]. More aggressive triple-negative breast cancers tend to occur more frequently in Black women; however, even within this particular subtype, five-year survival rates are lowest for Black women compared to other racial and ethnic groups [7]. Our institutional data reflects similar disparities. A higher percentage of Black patients in our study showed nodal involvement, a higher T stage, and a higher overall Stage compared to White or Hispanic patients, which are associated in prior studies with diminished survival. Our study also demonstrated more multifocal disease and bilateral disease in Black males. Stage 4 breast cancer was seen in a higher percentage of Black patients compared to other groups in our cohort, further underscoring the disproportionate burden of advanced disease in Black patients. National data support this pattern, with Black males demonstrating the lowest 1-year and 5-year survival compared to other races/ethnicities, with more frequent distant stage disease [8]. Distant-stage disease was also more commonly seen in Black males compared to males of other racial/ethnic groups [8]. Our study similarly found a higher prevalence of distant metastases in Black males, as two of our three patients with Stage 4 disease were Black.

Gynecomastia, a relatively common condition in men, represents the prevailing diagnosis in most diagnostic mammograms performed among symptomatic patients [5,9]. In healthy adult men, the incidence of clinical gynecomastia has been reported to be as high as 36%, and in a study of 100 unselected male autopsies, 55 cases of gynecomastia were identified, none of which had been evident clinically [10,11]. Often, a clinical or mammographic diagnosis of gynecomastia will obviate concerns for male breast cancer [2]. However, the role of gynecomastia as a risk factor for future cancers is not well understood, though both gynecomastia and male breast cancer represent sequela of high serum estrogen [12]. A pathologic review of 70 surgical cases in 1978 revealed that 40% of specimens contained microscopic gynecomastia [13]. In a meta-analysis of 11 case–control and 10 cohort studies, Brinton et al. found an association of clinical gynecomastia with increased male breast cancer risk (OR = 9.78) [14]. In a European multisite case–control study, Guenel et al. demonstrated a similar association (OR = 23.42) [15]. The exact relationship between clinical gynecomastia and male breast cancer has been difficult to discern, given the recall bias in patients with known cancers [15,16]. In our cohort of 26 males with breast cancer, the majority (69%) demonstrated gynecomastia on imaging. Although the small sample size precludes statistical comparison across demographics, gynecomastia was present in the majority of patients across all racial groups (71% of Black patients, 75% of Hispanic patients, and 63% of White patients). Notably, in our patients with bilateral breast cancer, both demonstrated gynecomastia (100%), and four out of five (80%) of our patients with multifocal disease demonstrated gynecomastia. This observation raises the possibility that multifocal or bilateral disease may be more frequently seen in the setting of gynecomastia, although this requires confirmation in larger cohorts. Currently, no strong consensus exists regarding the utility of routine breast cancer screening in patients with gynecomastia. These observations highlight a population that may warrant further study to better define the potential role of targeted screening strategies. These findings, when considered alongside existing guideline recommendations and national outcome data, suggest that further investigation into targeted screening strategies for men with gynecomastia may be warranted. This may be particularly beneficial for Black males, where health care disparities are likely present, and may help to mitigate disparities in outcomes.

Gynecomastia occurs across a wide range of age groups, from 21 to 81 years old, with peak periods noted in adolescence due to pubescent surges in estradiol and in men over the age of 65, generally attributed to hormonal shifts from testicular failure and age-related weight gain [5,12]. In all age groups, various drugs are known to cause gynecomastia, including marijuana products, spironolactone, diazepam, exogenous estrogen, androgens, digitalis preparations, thiazides, antiretroviral therapies, tricyclic antidepressants, and others [5]. Diseases commonly associated with gynecomastia include Klinefelter’s syndrome, chronic kidney disease, and chronic liver disease, with associations also seen with Sertoli and Leydig cell testicular tumors, resolving malnutrition, thyroid dysfunction, and adrenal insufficiency [5].

As discussed, gynecomastia is often diagnosed when there exists a clinical concern for male breast cancer. Male breast cancer accounts for 1% of all breast cancer cases. Among Black populations in the United States, this figure increases to 1.4%, and within sub-Saharan African populations, it can be as high as 14% [12,16]. Men with breast cancer tend to present at an older age, with a median age of 63, with clinically advanced tumors compared to women, on average demonstrating larger tumor size, greater likelihood of axillary nodal metastases, higher disease stage, and shorter overall survival [2]. While average-risk males have a 0.1% lifetime risk of breast cancer, BRCA1 mutation carriers have a 2% risk, and BRCA2 carriers have an 8% risk [2]. As with gynecomastia, male breast cancer is associated with hormonal shifts, with increased risk seen with hypoandrogenic conditions such as Klinefelter syndrome, testicular trauma, and infertility, as well as in hyperestrogenic conditions, such as hepatic disease [11].

Mammography is the preferred modality for the three imaging variants of gynecomastia (i.e., nodular, dendritic, and diffuse), with ultrasound evaluation largely considered noncontributory [2,17]. In cases where imaging findings raise suspicion for cancer, however, ultrasound has high specificity and enables targeted imaging of the axilla [2]. Mammography additionally represents the gold standard for diagnosing male breast cancer, with a high sensitivity (up to 100%) and specificity (up to 96%) [2]. Compared with female breast cancers, male breast cancers are less likely to appear spiculated and calcified [17]. Typically, male breast cancer manifests as a subareolar mass eccentric to the nipple. Mammographically dense breast parenchyma, which can be seen in males with gynecomastia, may mask an underlying cancer. Additionally, it is vital that the identification of gynecomastia mammographically not lead to perceptual errors, such as satisfaction of search, where cognitive attention is diverted to the gynecomastia, leading to missed diagnosis of coexisting breast cancer.

Current male breast cancer screening guidelines include the 2020 American Society of Clinical Oncology Guideline for Management of Male Breast Cancer, which recommends ipsilateral yearly mammography for males with a history of lumpectomy for breast cancer, regardless of genetic predisposition, as well as optional contralateral yearly mammography in men with a history of breast cancer and genetic risk [2]. Additionally, for BRCA-positive men, the 2023 National Comprehensive Cancer Network recommends a yearly clinical exam after age 35 and annual optional mammography in men with gynecomastia after age 50 or 10 years before the earliest known family history of male breast cancer [2]. Currently, ACR guidelines recommend yearly screening mammography for patients 40 or older with 5 years or more of hormone treatment [18]. This guideline may potentially extend to men with long-standing gynecomastia, given its hormonal basis. This is particularly important for Black males who may have worse outcomes, which may inform future efforts aimed at understanding and addressing observed disparities. These demographic disparities are further compounded by the gender gap in breast cancer outcomes between males and females. The prognosis for male breast cancer is worse compared to female breast cancer, with a significantly higher mortality rate. In a study spanning 11 years consisting of data from 16,025 males and 1,800,708 females, the 5-year survival for men is 77.6% compared to 86.4% for women [19]. A higher percentage of men present with advanced disease compared to women, with 5.8% of men presenting with stage IV disease compared to 3.8% of women [19]. Even after full adjustment for contributing factors, male gender was an independent factor influencing poorer prognosis with 15% higher 3-year mortality and 18% higher 5-year mortality compared with female counterparts, which could not be explained by the general excess mortality of men compared to women [19]. This difference was most dramatic in ER+ breast cancer, where the Hazard Ratio was higher for the first 3 years after cancer diagnosis for Stage I or Stage II ER+ breast cancer, while no difference in mortality was seen with ER- disease except in Stage IV disease [19]. Existing screening recommendations and guideline frameworks may provide a foundation for future studies examining whether tailored screening approaches could impact gender and demographic disparities

Our institutional findings should be interpreted as descriptive and hypothesis-generating and are not intended to establish statistically significant differences or causal relationships. This study was not designed to evaluate screening efficacy or outcomes, and no screening recommendations can be derived from these data.

### Limitations

This study was a single-institution, observational analysis aimed at presenting our institution’s experience with predominantly underrepresented male patients presenting with breast cancer in our community. This study has several limitations. Its retrospective, single-institution design introduces potential selection and referral biases, as the cohort includes only male patients who underwent mammographic evaluation at a tertiary care center and may not represent the broader Bronx population. Patients referred for diagnostic imaging may have had higher clinical suspicion, potentially overestimating cancer prevalence and disease stage. Survivorship bias is also possible, as patients with rapidly progressive disease who did not survive to imaging or biopsy were not captured. Information bias may result from incomplete or inaccurate documentation in the medical record, particularly regarding clinical risk factors and staging data. Confounding by unmeasured factors—including age, genetic predisposition, comorbidities, socioeconomic status, and access to care—cannot be excluded, especially as no multivariable adjustment was performed. Misclassification bias is possible due to reliance on medical record documentation for clinical variables and staging data, as well as imaging-based assessment of gynecomastia; however, consensus review by two experienced breast imagers mitigated the latter. Given these limitations and the small sample size, findings are descriptive and should be validated in larger, prospective studies.

We acknowledge the absence of statistical analysis as a limitation of this study, due to the small sample size and resulting constraints on statistical power. Furthermore, we examined a cohort diagnosed during a specific time period, and the lack of available longer-term clinical data restricted our ability to perform comprehensive survival analysis. Future studies with larger cohorts and longer follow-up are needed to evaluate survival differences across demographic groups.

## 5. Conclusions

In this single-institution retrospective cohort, Black male patients were observed to present more frequently with higher-stage disease and with multifocal or bilateral breast cancer compared with other demographic groups; however, these findings are descriptive and should be interpreted cautiously. Black males have been reported to experience poorer prognosis relative to males of other racial groups [8], and males overall have poorer outcomes compared with females. Gynecomastia was commonly present among males with breast cancer across all demographic groups in our cohort. These observations highlight populations that may warrant further study to better understand disease presentation and to inform future research on early detection strategies. Larger, prospective studies are needed to evaluate potential screening approaches and to determine whether targeted interventions could impact observed gender and demographic differences in outcomes.

## Figures and Tables

**Table 1:** Patient characteristics and clinical presentation.

Patient Number	Gynecomastia	TNM	Race/Ethnicity	Survival	Multifocal and/or Bilateral	Receptor Type	Genetic Testing	Family History (Cancer)	Risk Factors	Clinical Presentation	Mammogram (Screening vs. Diagnostic) at Presentation	Comorbidities
01	Yes	T2N1M0	Black	No (2Y)		ER+	Negative	Prostate cancer in father, cervical cancer in paternal grandmother, prostate cancer in half-brother.	No	Palpable left breast mass with a palpable left axillary nodule	Diagnostic	N/A
02	No	T1N0M0	Black	Yes (8Y)		ER+	None found in chart	None	Former smoker	Palpable left breast mass	Diagnostic	ESRD on dialysis, HTN, T2DM
03	No	T1N0M0	Black	Yes (9Y)		ER+	VUS in BRIP1	None	History of obesity; former smoker	Palpable right breast mass with spontaneous, bloody right nipple discharge	Diagnostic	T2DM, HTN
04	Yes	T3N1M0	Black	Yes (8Y)	Multifocal/Bilateral	ER+	Declined genetic testing	Lung cancer in mother	History of obesity	Right palpable breast mass	Diagnostic	HTN
05	Yes	T2N3M0	Black	No (1Y)		ER+	Negative	Lung cancer in maternal uncle, prostate cancer in maternal cousin, cancer of unknown type in maternal aunt	History of obesity; former smoker	Right breast mass	Diagnostic	HTN
06	Yes	T2N1M0	Black	Yes (5Y)		ER+	VUS in MUTYH	Colon cancer in mother; prostate cancer in father; stomach cancer in sister; prostate cancer in uncle	History of obesity; former smoker	Left breast mass	Diagnostic	HTN, questionable history of alcohol abuse; hepatitis 2/2 to either fatty or alcohol
07	Yes	T1N0M0	Black	Yes (4Y)		ER+	None found in chart (Patient was referred but has not pursued testing as of today)	Breast cancer in grandmother (unknown type or paternal/maternal origin but then in chart later denies breast cancer family history); lung cancer in maternal grandmother	No	Right breast mass	Diagnostic	No
08	Yes	T1N0M0	Black	Yes (3Y)	Multifocal	ER+	None found in chart (Patient was referred but has not pursued testing as of today)	Prostate cancer in brother; breast cancer in sister (*n* = 2); lymphoma in brother; colon cancer in sister; breast cancer in nephew; breast cancer in niece	History of cancer (right clear cell renal cell carcinoma s/p nephrectomy and prostate cancer s/p seed implants); former smoker	Surveillance imaging for RCC with noted left breast FDG activity	Diagnostic	T2DM, HTN
09	No	T2N1M0	Black	Yes (3Y)		ER+/HER2+	Negative	None	Active smoker	Pain in left arm and then noted mass in left breast	Diagnostic	HTN
10	Yes	T3N3M1	Black	No (2Y)		ER+	TP53 C238Y, GATA3 T441fs, ATM R3008H, JAK2 V617F, VUS in VHL V165I, VUS in NF1 F1413del, TP53 R248W	Prostate cancer in brother; breast cancer in first-degree sibling (unclear which sibling due to chart inconsistency)	Former smoker; history of cancer (prostate)	Palpable left breast mass	Diagnostic	HTN, Sickle cell trait
11	No	T4bN3M1	Black	Yes (2Y)		ER+/HER2+	Negative	Prostate cancer in father	None	Left breast mass fungating through the skin	Diagnostic	No
12	Yes	T2N0M0	Black	No (0.5Y)		ER+	BRCA2	Breast cancer in sisters (*n* = 4), brother with prostate cancer, paternal cousin with prostate cancer; father with gi cancer; paternal grandmother with breast cancer	BRCA2	Palpable right breast mass	Diagnostic	HTN
13	Yes	T2N1M0	Black	Yes (1Y)	Multifocal	ER+	Negative	Breast cancer in daughter	Former smoker; history of obesity	Palpable left breast mass	Diagnostic	T2DM, HTN
14	Yes	Tis	Black	Yes (1Y)	Bilateral	ER+	Negative	Unknown (adopted)	Former smoker; history of obesity	Palpable right breast mass with nipple discharge	Diagnostic	HTN, T2DM
15	Yes	T2N1M0	Hispanic	Yes (9Y)		ER+	BRCA2	Skin cancer in father; breast cancer in sister; breast cancer in maternal cousin	Former smoker; BRCA2	Palpable right breast mass with nipple retraction	Diagnostic	HIV; Paget’s Disease
16	Yes	T2N0M0	Hispanic	Yes (8Y)		ER+	VUS in CHEK2, VUS in NBN	None	History of obesity	Palpable left breast mass	Diagnostic	T2DM, HTN
17	No	T1N0M0	Hispanic	Yes (9Y)		ER+	Negative	None	History of cancer (prostate)	Palpable left breast mass	Diagnostic	No
18	Yes	T2N1M0	Hispanic	Yes (1Y)		ER+/HER2+	RAD51C (EX4del)	Prostate cancer in father, stomach cancer in mother, breast cancer in maternal grandmother, brain cancer in maternal aunt, stomach cancer in maternal cousin, breast cancer in paternal aunt	Former smoker; RAD51C gene +	Palpable left breast mass with a palpable left axillary nodule	Diagnostic	No
19	No	T2N0M0	White	No (4Y)		ER+	None found in chart (patient was referred but was unable to pursue by time of death)	Breast cancer in maternal aunt; cancer (unknown) in maternal grandmother	Ashkenazi Jewish decent	Induration below right nipple	Diagnostic	T2DM, HTN, CKD
20	No	T1bN1aM0	White	Yes (7Y)	Multifocal	ER+	None found in chart (patient was referred but has not pursued testing as of today)	Family history of breast cancer (further details unknown)	History of obesity; history of cancer (prostate); declined endocrine therapy (tamoxifen	Yearly screening mammogram due to hx of breast cancer s/p lumpectomy w/IORT, local repair, and SLNBx in 2015	Screening	No
21	Yes	T4N3M1	White	No (2Y)		ER+	Negative	Lung cancer in half-sister; prostate cancer in father; leukemia in half-brother	History of obesity; former smoker	Palpable right breast mass with nipple inversion	Diagnostic	T2DM, HTN
22	Yes	T2N1M0	White	Yes (3Y)		ER+	BRCA2 (also possibly JAK2, CDH1, PMS2, SMAD4, CCND1 but unclear due to chart documentation)	Breast cancer in maternal aunt (mother’s fraternal twin); three paternal aunts with breast cancer	Ashkenazi Jewish descent; BRCA2	Palpable, tender right breast mass	Diagnostic	No
23	Yes	T1N0M0	White	Yes (3Y)		ER+	Negative	None	Ashkenazi Jewish descent	Palpable left breast mass	Diagnostic	Ulcerative Colitis, Prothrombin gene mutation
24	Yes	T1N0M0	White	Yes (3Y)		ER+	CHEK2	Colon cancer in mother; lymphoma in mother, breast cancer in twin brother, melanoma in twin brother, cancer (unknown) in paternal aunt	Ashkenazi Jewish descent; history of obesity; former smoker; CHEK2 gene	Annual screening given high risk of breast cancer	Screening	HTN
25	Yes	TisN0M0	White	Yes (3Y)		ER+	Negative	Breast cancer in daughters (*n* = 2)	Ashkenazi Jewish descent; history of obesity; history of cancer (lambda light chain myeloma and prostate cancer s/p RT);	Palpable left breast mass with a palpable left axillary nodule	Diagnostic	CKD, HTN
26	No	T1N0M0	White	Yes (1Y)		ER+	Negative	Breast cancer and malignant melanoma in mother; pancreatic cancer in father, breast cancer in maternal grandmother, cancer (unknown) in maternal grandfather	Ashkenazi Jewish descent	Palpable right breast mass	Diagnostic	No

**Table 2:** (**A**) Multifocal and bilateral disease. (**B**) Proportion of multifocal and/or bilateral disease in each group.

(**A**)				
	**Multifocal Only**	**Bilateral Only**	**Multifocal and Bilateral**	**Multifocal and/or Bilateral**
**Number of Patients**	3	1	1	5
**Race/Ethnicity**				
Black	2 (75%)	1 (100%)	1 (100%)	4 (80%)
White	1 (25%)	0	0	1 (20%)
Hispanic	0	0	0	0
(**B**)				
**Race/Ethnicity**	**Multifocal/Bilateral**		**Total Patients in Cohort**	**Proportion**
Black	4		14	28.60%
White	1		8	12.50%
Hispanic	0		4	0%

## Data Availability

The data presented in this study are available on request from the corresponding author. The data are not publicly available due to privacy and ethical restrictions.

## Abbreviations

The following abbreviations are used in this manuscript:

IRB	Institutional Review Board
HIPAA	Health Insurance Portability And Accountability
CT	Computed tomography
MLO	Mediolateral oblique
COVID-19	Coronavirus disease 2019
pCR	Pathologic complete response
OR	Odds ratio
ACR	American College of Radiology
